# Improving obesity management training in family medicine: multi-methods evaluation of the 5AsT-MD pilot course

**DOI:** 10.1186/s12909-019-1908-0

**Published:** 2020-01-07

**Authors:** Thea Luig, Sonja Wicklum, Melanie Heatherington, Albert Vu, Erin Cameron, Doug Klein, Arya M. Sharma, Denise L. Campbell-Scherer

**Affiliations:** 1 Office of Lifelong Learning & Physician Learning Program, University of Alberta, Edmonton, Canada; 2 Cummings School of Medicine, University of Calgary, Calgary, Canada; 3 Core Internal Medicine, University of Alberta, Edmonton, Canada; 4 Department of Human Sciences, Northern Ontario School of Medicine, Sudbury, Canada; 5 Department of Family Medicine, University of Alberta, Edmonton, Canada; 6 Department of Medicine, Division of Endocrinology, University of Alberta, Edmonton, Canada; 7 Alberta Diabetes Institute, University of Alberta, Edmonton, Canada; 8 2-590 Edmonton Clinic Health Academy, Office of Lifelong Learning/Physician Learning Program, University of Alberta, Edmonton, AB T6G 1C9 Canada

**Keywords:** Obesity, Education, medical, Primary health care, Evaluation study

## Abstract

**Background:**

Quality, evidence-based obesity management training for family medicine residents is needed to better support patients. To address this gap, we developed a comprehensive course based on the 5As of Obesity Management™ (ASK, ASSESS, ADVISE, AGREE, ASSIST), a framework and suite of resources to improve residents’ knowledge and confidence in obesity counselling. This study assessed the course’s impact on residents’ attitudes, beliefs, and confidence with obesity counselling.

**Methods:**

The course combines lectures with a bariatric empathy suit experience, standardized and in-clinic patient practice, and narrative reflections. Using a multi-methods design we measured changes in 42 residents’ attitudes, beliefs, and self-confidence and thematically analyzed the narrative reflections to understand residents’ experience with the course content and pedagogy.

**Results:**

Following the course, residents reported improved attitudes towards people living with obesity and improved confidence for obesity counselling. Pre/post improvement in BAOP scores (*n* = 32) were significant (*p < .001)*., ATOP scores did not change significantly. Residents showed improvement in assessing root causes of weight gain (*p* < .01), advising patients on treatment options (*p* < .05), agreeing with patients on health outcomes (*p* < .05), assisting patients in addressing their barriers (*p* < .05), counseling patients on weight gain during pregnancy, (*p* < .05), counseling patients on depression and anxiety (*p* < .01), counseling patients on iatrogenic causes of weight gain (*p* < .01), counseling patients who have children with obesity (*p* < .05), and referring patients to interdisciplinary providers for care (*p* < .05).

Qualitative analysis of narrative reflections illustrates that experiential learning was crucial in increasing residents’ ability to empathically engage with patients and to critically reflect on implications for their practice.

**Conclusion:**

The 5AsT-MD course has the potential to increase residents’ confidence and competency in obesity prevention and management. Findings reflect the utility of the 5As to improve residents’ confidence and competency in obesity management counselling.

## Background

The prevention and management of obesity and related chronic diseases is an integral aspect of family medicine. These conditions are affecting increasing numbers of adults and children [1, 2]. Both the United States Preventive Services Task Force and the Canadian Task Force on Preventive Health Care recommends that primary care practitioners screen patients, counsel regarding weight loss, and refer to structured behavioural interventions aimed at weight loss [3, 4]. However, addressing obesity in a clinical consultation can be challenging for both physicians and patients. In fact, many physicians are not routinely discussing weight and report a lack of confidence in obesity management skills, lack of time, and fear of endangering their relationship to patients by discussing weight [5–11]. Predominant societal negative attitudes towards persons living with obesity also affect care and further complicate clinical conversations [12, 13]. As a result, patients often feel uncomfortable bringing up weight concerns despite expressing their need for physicians to initiate such a conversation [14–17].

One of the challenges for effective obesity counselling is that medical residents and students do not receive sufficient training on the complex biopsychosocial etiology of obesity and evidence-based management strategies, and these topics are not well covered in medical exams [18–22]. A recent review concluded that across the world there is a paucity of obesity education programs for learners in health professions [23]. Reasons include the relative newness of classifying obesity as a disease, the complexity of causation and management, and the socio-cultural and personal associations that physicians, residents, medical educators, and patients bring to each interaction that addresses obesity [13, 24–27]. The result is a substantial lack of capacity in primary health care to deliver comprehensive, tailored, and effective obesity prevention and management [28–31]. Despite calls from international health and professional organizations to improve curriculum and training in obesity and evidence that high-quality education programs can improve outcomes [23], progress has been slow and no widely recognized courses have been implemented to date [30, 32–34].

It is essential that family medicine residents are prepared to: 1) identify complex causes of obesity and its associations with comorbidities and mental health, 2) counsel on appropriate weight management options, and 3) navigate interdisciplinary teams and resources to best assist the patient. There is a pressing need to create high quality, evidence-based obesity management training courses for family medicine residents so they can deliver quality patient care.

This paper reports on the pilot run of a new educational intervention to better prepare family medicine residents for obesity management, the 5AsT-MD course. The course draws on the “5As of Obesity Management™”(ASK, ASSESS, ADVISE, AGREE, ASSIST), which is an evidence-based framework to guide practitioners’ obesity counseling [35–37], and utilizes available 5AsT tools and resources developed by the 5As Team to support primary care obesity conversations [38]. In addition to introducing these counseling supports, the course aimed at fostering transformative learning through combining interactive lectures with experiential learning including wearing bariatric empathy suits, patient practice, and reflection. The purpose of the study was to understand the courses’ impact on residents’ knowledge, attitudes, and confidence with obesity counselling.

## Methods

The 5AsT-MD course was delivered to two cohorts (fall 2015 and spring 2016) of first year family medicine residents training at the University of Alberta (*n* = 61) as part of the mandatory Doctor-Patient Relationship (DPR) course. Course elements are described in detail in Table 1.

**Table 1:** The 5AsT-MD Program

The 5AsT-MD course is designed to be practical and adaptable to different educational settings and needs. The fall cohort completed the following course components in eleven hours over two days. The spring cohort completed the same content in eight hours over two days.	
Course component	
1	Interactive, discussion-based lectures covering: 1) the complex etiology of obesity and its chronicity, 2) an introduction to the 5A’s of Obesity Management and the 5AsT approach, 3) assessment and management of obesity in pediatrics 4) prevention, pregnancy and postpartum, 5) management of obesity, including lifestyle changes, medications and bariatric surgery.
2	Empathy suit experience: residents are given an opportunity to wear a empathy suit, which simulates a body size in the obesity class. Learners experienced the incumberance of obesity spending approximately 15 min in a Smart Condo executing tasks of daily living (i.e., getting dressed, cleaning the apartment, getting out of bed, making the bed). The university provides these resources as part of the Health Sciences Education and Research Commons. Empathy suits are available commercially, and the tasks of daily living exercise can be modified to other local circumstances [39–41].
3	Following this exercise, residents are asked to complete a one-page narrative reflection on their experience wearing the suit. At the next session, residents discuss their experiences and reflections in small groups facilitated by expert preceptors.
4	Standardized patient interviews: Residents demonstrate their use of the 5A’s by practicing with standardized patients. Patient cases were designed to focus on specific parts of the 5A’s (i.e., ASK, ASSESS, ADVISE, AGREE, ASSIST) and to allow residents to practice the skills and tools they have learned.
5	Following this exercise, the residents debrief in small groups, which include their preceptor, the standardized patient, and their peers.
6	In-clinic practice: Residents practice the newly acquired skills and knowledge with one of their own patients in clinic.
7	Residents reflect on their experience in a one-page narrative, which they debrief with their preceptor.

In summary, the 5As approach to obesity management understands root causes of obesity as more than diet and exercise, and considers the impact of mental health, social situations, obesogenic medications, and comorbid diseases on peoples’ ability to change. The course drew on transformative pedagogy that emphasizes critical reflection and experiential learning [42]. The 5As approach and tools were reviewed in the interactive lectures and practiced both with standardized patients during the course and with patients in clinic. Because of time restrictions through departmental course restructuring, we condensed and refined some of the lecture material and optimized scheduling of the activities for the second cohort using feedback from the first cohort.

We used a multi-methods research design to assess: 1) Did the 5AsT-MD course improve attitutdes and beliefs by increasing awareness of the complexity and lived reality of obesity? 2) Did the 5As approach and 5AsT resources improve residents’ confidence in their obesity counselling practice? All residents, regardless of whether they consented to participate in the research study, were asked to complete the course assignments (questionnaires and narrative reflections) which were not graded. Written consent was obtained from residents before the first lecture of the course. These assignments were de-identified by a neutral third party. Researchers were only given access to data from residents who consented.

### Quantitative methods: beliefs, attitudes, and confidence measures

Residents completed the Beliefs About Obese Persons Scale (BAOP), and Attitudes Towards Obese Persons Scale (ATOP) prior to and immediately following the course. Both are brief and well validated measures that made participation in an evaluation more feasible for our residents cohort. The BAOP is a continuous scale, derived from eight Likert Scale questions, that measures beliefs about the causes of obesity [43]. Scores range from 0 to 48 with higher scores suggesting a stronger belief that an individual may not control and not be responsible for their disease, as compared to the inverse of blaming or assigning fault to the individual. Previous research has reported adequate internal reliability (Chronbach’s α = 0.65 to 0.82) [44].

The ATOP is also a continuous scale, derived from 20 Likert scale questions measuring perceptions and attitudes about persons living with obesity. Scores range from 0 to 120 with higher scores reflecting more positive attitudes. Previous research has reported adequate internal reliability (Chronbach’s α = 0.76 to 0.84) [43].

Changes in residents’ level of confidence was assessed using a 29-item questionnaire which was developed specifically for this course (see additional file 1). The questionnaire collects demographic information (age, gender, years of medical training), and uses a 5-point Likert scale to rate: 1) the importance of obesity management as part of family physicians’ role; 2) perceptions on the adequacy of previous training in obesity management; 3) movitation to learn more about this area; and, 4) 22 items about comfort using the 5As in their consultations with patients.

Data from participants who completed all three pre- and post- questionnaires were included in the analysis. Mean scores and standard deviations were calculated for each questionnaire. Two tailed, paired *t-tests* were applied, in order to assess change between pre and post using Microsoft Excel 2010. *P* values less than 0.05 were considered statistically significant.

### Qualitative methods: narrative reflections

To facilitate critical reflection, learning, and foster sharing and support among plural - residents, participants wrote two brief narrative reflections as part of their course assignment: one after wearing the empathy suit; and the second after the in-clinic practice. To mitigate challenges with residents’ ability to reflect on their experience, revealed through preliminary analysis of the fall cohort, we added guiding questions to the instruction sheets for the spring cohort (see additional file 2).

Narratives of consenting residents were analyzed to gain insights into their experience of the course content and pedagogy. Narratives were de-identified and imported into NVivo11 for thematic analysis. To capture insights not anticipated by the research team and literature, TL first coded inductively and noted emerging patterns. Analyst triangulation was used in two steps. First, data, codes, and emergent themes were discussed during monthly team meetings that included researchers, course instructors, and a patient champion until consensus was reached. Second, guided by these patterns, TL and EC reviewed the literature in education and added theoretically derived codes to the node manual to generate findings that can be analyzed and situated in existing pedagogical theory. All narratives were re-coded using the revised manual including inductive and deductive nodes. Because logistical changes were made to the course from fall to spring cohorts, we compared between cohorts as well as comparing first and second reflections within cohorts to crystallize patterns and themes.

## Results

### Demographic data

Written consent was obtained from 42 (69%) of the 61 residents who were enrolled in the DPR course from the fall and spring cohort. Demographic characteristics are detailed in Table 2. Of the 42 residents who consented, 32 completed all three questionnaires. All 42 residents submitted a narrative reflection on their experience with the empathy suit and 31 residents submitted a narrative reflection based on their experience with a patient in clinic.

**Table 2:** Demographic characteristics of family medicine residents (*n* = 42)

Age	*N*	%
20–25	12	28.6
26–30	25	59.5
31–35	3	7.1
40+	1	2.4
Missing data	1	2.4
Gender		
Male	18	42.9
Female	24	57.1
Years of medical training		
3	4	9.5
4	23	54.8
5	10	23.8
6+	5	11.9

### Changes in beliefs, attitudes, and confidence

Mean scores on the BAOP questionnaire revealed a significant improvement in study participants’ positive beliefs about people living with obesity following the course. ATOP questionnaires which started out high, yielded no meaningful change in attitudes toward people living with obesity (see Table 3).

**Table 3:** Differences between BAOP and ATOP scores pre- and post- course (*n* = 32)

	Pre-course		Post-course		95% Confidence Intervals	t-test	df	Sig. (2-tailed)
	M	SD	M	SD				
BAOP score	19.86	5.94	24.03	7.54	−4.77 to −1.35	−3.65	31	.001
ATOP score	73.15	16.58	69.26	17.75	−0.58 to 10.40	.62	31	.0959

*BAOP* Belief About Obese Persons Scale, *ATOP* Attitudes Towards Obese Persons Scale

Prior to the course, all of the residents who submitted questionnaires (*n* = 32) believed that obesity management was an important part of their job as a physician, 28% felt that they had received adequate medical training to manage obesity, and 91% were motivated to learn more about the topic (see Table 4). Following the course, residents still felt that obesity management was an important part of their job, but 47% of the residents felt better trained and 88% wanted to learn more.

**Table 4:** Resident perceptions on obesity management importance and training. (*n* = 32)

		Pre-course	Post-course
		%	%
Obesity management is an important part of my job as a family physician	Strongly Agree	59.4	62.5
	Agree	40.6	37.5
My medical training before this session has adequately prepared me to understand and manage obesity with patients	Strongly Agree	3.1	6.3
	Agree	25.0	40.6
	Neutral	21.9	28.1
	Disagree	50.0	21.9
	Strongly Disagree	0	3.1
I am motivated to learn more about the effective prevention and management of obesity	Strongly Agree	46.9	31.2
	Agree	43.8	56.3
	Neutral	9.4	12.5

Statistically significant results were found in 9 of the 22 parameters on the course questionnaire, which measured changes in residents’ self-reported confidence in their weight management encounters (see Table 5). Following the course, residents felt more comfortable assessing root causes, advising on treatment options, agreeing with patients on goals, assisting patients in addressing barriers, counseling on weight gain during pregnancy, counseling on weight-related depression and anxiety, counseling on iatrogenic causes of weight gain, counseling patients who have children with obesity, and referring patients to interdisciplinary healthcare providers for care.

**Table 5:** Differences between residents’ self-reported confidence pre- and post- course (*n* = 32)

Questions	Pre-Course		Post-Course		95% Confidence Intervals	t	df	Sig. (2-tailed)
	M	SD	M	SD				
1. Asking for a patient’s permission to talk about his/her weight.	2.19	1.00	1.88	1.07	−0.12 – 0.75	1.47	31	.152
2. Assessing a patient’s obesity-related risks and complications.	1.88	0.83	1.72	0.63	−0.88 – 0.40	1.31	31	.201
3. Assessing a patient’s potential root causes of weight gain.	2.47	0.95	1.97	0.54	0.15–0.85	2.89	31	.007
4. Advising patients on obesity-related risks and complications.	1.91	0.82	1.84	0.68	−0.24 – 0.37	0.42	31	.677
5. Advising patients on available treatment options for obesity.	2.63	1.04	2.19	0.82	0.07–0.80	2.44	31	.021
6. Advising patients on long-term strategies to manage weight.	2.59	0.98	2.28	0.77	−0.07 – 0.69	1.67	31	.106
7. Agreeing with patients on realistic weight-loss expectations.	2.13	0.79	1.84	0.81	−0.06 – 0.63	1.66	31	.107
8. Agreeing with patients on sustainable behavioural/lifestyle goals.	2.13	0.79	1.91	0.59	−0.07 – 0.50	1.56	31	.129
9. Agreeing with patients on goals for health outcomes.	2.19	0.78	1.81	0.69	0.07–0.68	2.55	31	.016
10. Assisting patients in addressing their barriers to proper weight management.	2.41	0.95	1.94	0.76	0.08–0.86	2.46	31	.020
11. Providing education and resources to encourage patients’ self-management.	2.44	1.01	2.13	0.75	−0.12 – 0.75	1.47	31	.152
12. Counseling patients on physical activity and weight control.	2.16	0.95	2.13	0.87	−0.32 – 0.38	0.83	31	.856
13. Counseling patients on appropriate weight gain during pregnancy.	2.25	1.11	1.84	0.68	0.03–0.78	2.20	31	.035
14. Counseling patients on emotional eating.	3.06	1.11	2.75	1.08	−0.15 – 0.77	1.38	31	.177
15. Counseling patients on weight-related depression and anxiety.	3.06	1.01	2.50	0.92	0.20–0.93	3.14	31	.004
16. Counseling patients on iatrogenic causes of weight gain.	2.56	0.98	2.09	0.69	0.10–0.83	2.61	31	.014
17. Counseling patients who have children with obesity.	3.22	1.16	2.72	1.08	0.08–0.92	2.43	31	.021
18. Addressing differences that may come up in your consultation due to culture or beliefs.	3.03	0.97	2.75	0.92	−0.16 – 0.72	1.30	31	.203
19. Addressing weight gain with patients who have multiple co-morbidities.	2.34	0.87	2.16	0.68	−0.12 – 0.50	1.24	31	.226
20. Discussing weight with patients who have a family history of obesity.	2.31	0.86	2.22	0.71	−0.30 – 0.49	0.49	31	.629
21. Discussing weight and lifestyle management with patients who are at risk of obesity.	2.25	0.88	2.06	0.72	−0.12 – 0.50	1.24	31	.226
22. Referring patients with obesity to the appropriate healthcare provider for care.	2.38	1.01	2.03	0.65	0.05–0.64	2.35	31	.025

Legend: pre vs. post comparison with paired *t*-test

1 = Very Comfortable, 5 = Very Uncomfortable

### Narrative reflections on course experience and utility for practice

Overall, residents perceived the teaching content and methods as useful and offering value for their practice. The majority expressed appreciation for the experiential elements of the course. Four themes emerged during analysis; representative quotes are given in Table 6.

**Table 6:** Representative quotes for the four themes of the qualitative analysis

1. Empathy and resistance	
*Unexpected emotional responses*: (1) At one point I glimpsed myself in the mirror and I could hardly recognize myself. I admit I am ashamed that I felt disgusted at how I looked. (R 31) (2) While I expected to find the household chores more tiring, I was surprised by how self-conscious I actually started to feel while wearing the empathy suit (even just for a few minutes). I have always been a small person and I almost felt a sense of embarrassment while wearing the suit. (R 4)	
*Physical aspect*: (1) Going through the different activities made me extremely breathless and insecure at every point of the way where I was unable to see my own feet and not knowing where I am stepping. I was extremely scared to even step into the bathtub! Let alone gathering courage to go out to a swimming pool to get some exercise!! (R 52) (2) After this experience, it is much easier to sympathize with the reluctance to exercise. When every little movement is difficult, painful and requires a significant effort, why would anyone be motivated to do any additional physical activity? (R 35)	
*Mental aspect: I think the more difficult thing for me to think about was looking in the mirror with the suit on. I felt pretty awful and would hate if I ever ended up with a weight like that. It really determined the superficial aspect of being overweight. (R 15)*	
*Empathy and re-thinking counselling practice*: Prior to this eye opening experience, I felt I had some good knowledge about obesity and I am comfortable talking to my patients about their weight, to offer them evidence based weight loss strategies. I felt it was just a matter of setting up goals, keep pushing themselves to stay active and maintain a good diet for the weight loss to occur. It was difficult for me to put myself in their shoes and see the physical limitations they have with their body habitus. It felt like a workout to me just doing activities of daily living in the twenty minutes I was wearing the empathy suit, which only weighted 10lbs. I now start to see how silly some of my recommendations were. I am able to better sympathize with my patients and will think of advice that is more achievable and realistic for them. (R 36)	
*Resistance*: My predominant feeling is one of annoyance and frustration with regards to this experience. (R 13)	
2. Reflexivity: weight counselling practice and role identity	
*Complexity*: As now I have realized, obesity is not unlike arthritis or atherosclerosis in that it is often challenging enough to halt its progression. I used to consider it as a will-influenced reversible process, but now I realize obesity is often impossible to modify when there are multiple resistant contributing factors. Without sustained lifestyle modifications, patients often yoyo through weight fluctuations with short term interventions and eventually become fed up with frustration and depression. That is what happened [to the patient they were counselling]. When a different physician preached her each time about simple concepts and empty slogans, without realistic management specifics, she only got reminded of her sufferings so far. (R 61)	
*Re-thinking assumptions*: I can admit I have made assumptions about people living with obesity. One of those assumptions is these individuals had a choice and it was their fault that they have gained weight. However, I have come to realize that the causes for obesity are multifactorial and rather complex. The exercise of wearing the empathy suit certainly reminded me of how obesity is a difficult health condition to live with and it is not as simple as losing some pounds by altering the energy in and energy out eq. (R 39)	
*Counselling practice*: (1) Reflecting on this experience, I don’t think I gave her [the in-clinic patient] the appropriate level of compassion and respect she deserved. Now that I think of it, she was just looking for an answer. An answer to the question, “Why do I weight more than most people when I never used to be this way?” Now that I think of it, if I had been in her position I would have been incredibly frustrated with her situation and the response from the medical system. (R 2) (2) Often, we dismiss the obese or “fat people” and say people should exercise more and eat less. It is difficult to understand how and why people become so obese. Perhaps, I am not as tolerant as I should be. Society and our choices don’t help either, when fast food is cheaper than fresh vegetables? When we are so rushed for time because we have to work fulltime in order to make ends meet? (R 31)	
*Role identity*: How can I support people thought this struggle and health challenge? What is my role? Where do I fit? (R 31)	
*Critique:* Please, there are more important and pressing things that I should be focusing on. (R 20)	
3. Utility of the 5As approach and 5AsT tools	
*Utility of the 5As*: (1) This framework of 5As will actually be useful in many settings to set an appropriate discussion. It is a great tool to have as a resource. (R 14) (2) I appreciate the tools provided to us at our session. It may seem intuitive but when I implement it into practice, it can be challenging. (R 22) (3) Being introduced to the approach to weight management via the 5AsT/4 M [4 M’s of obesity assessment] has provided me with a foundation of knowledge and practical tools that can assist me in supporting my patients better. (R29) (4) I am so happy to know [sic: now] have an approach and also one that I can do myself without having to refer the patient away. I know that I have that in my back pocket as extra help if things are not going well with my help alone. (R 15)	
*The importance of the “Ask”:* The most useful learning point for me was to preface any advice or discussion about weight by asking for the patient’s permission. This point really helps to make explicit the respect you have for patient decision making for their health. (R 35)	
*Self-efficacy:* (1) After reflecting on this encounter, I felt like I had a framework for the discussion, and could provide some realistic goals or expectations. The conversation still felt awkward, but I do think I’ll feel more confident in bringing up the issue of weight with patients in the future. (R 58) (2) On the whole, the encounter was encouraging for me as a physician because I feel like I can now at least START [original emphasis] the conversation about weight, even in children despite not always having the answers or solutions. I plan to take the resources I’ve been given and continue to practice having these conversations to become more proficient in obesity management. (R 24)	
*Mastery:* I have since been able to use the 5AsT tools for other patients and each time I feel the patient walks out happier than if I had just told them to eat less and move more. It’s the full discussion about weight and the underlying etiology of it that really helps a patient realize what some of their obstacles are, because honestly many of them are hard to pick out on your own. (R 15) This was a successful visit using the 5AsT tool and I will try to utilize it more in my clinical practice. (R 36)	
4. Challenges	
I feel like this symbolizes one of the largest challenges to discussing obesity and weight in the family practice; it is seldom that people book their appointments to chat only about weight, despite it being a topic that needs lengthy discussion. (R 6)	
We have all dealt with obese patients throughout our training, and experienced the difficulties of treating such patients. Physically it is more difficult to do physical exams, they often have more comorbidities, and we experience personal frustration with being unable to help them manage their obesity. I don’t think it is a bias to dislike treating obese patients because of these issues. […] I would just prefer if they were not obese as that would benefit their health, as well as make my job easier. Just like we become frustrated with patient that do not stop smoking, we also become frustrated with those that have been unsuccessful in controlling their weight. I think this is a natural reaction because we can see that such individuals are at greater risk for a variety of health problems in the future. As physicians this is something we obviously want to avoid. (R 12)	
I find weight loss difficult to discuss for two reasons. First, weight loss is challenging for patients. They don’t realize how difficult it is from a biological perspective to lose weight, and therefore their efforts seem to have no effect. Sometimes maintaining their weight can be a victory but patients don’t see it that way. Second, I question the futility of patient counselling regarding weight, I have nothing magical to offer them aside from diet and exercise except in extreme circumstances. (R 5)	
Personally, I find that I do have some biases in terms of patients with obesity. I had believed that any single patient could lose weight, as long as they were willing and motivated enough. Thus, when interacting with patients who were not motivated to increase their activity or change their diet, I would often be frustrated and thus become biased towards them. In terms of pure science and numbers, it is possible for every single patient to lose weight. Ensure calories consumed are less than calories expended will definitely lead to weight loss. However, this sort fo view does not consider the patient as a whole, or as a person. After the empathy simulation session, I find that I have become more empathetic to the plight of obese patient [sic]. (R 31)	

Legend: (R #) = Resident anonymized participant ID

### Experiential learning: increased empathy and evoked resistance

Experiential learning elements of the course proved crucial in increasing residents’ stated ability to empathically engage with patients and critically reflect on the implications for their practice. The empathy suit experience emotionally impacted residents who did not have previous lived experience with overweight or obesity. This helped them examine their assumptions about living with obesity. Most noted surprise about how cumbersome tasks of daily living were in the empathy suit. They described feeling exhausted, breathless, afraid of not being able to get out of bed, insecure about falling, and wanting to avoid unnecessary energy expenditure. Many wrote about how the experience of imagining themselves in a larger body, caused feelings of shock, shame, self-consciousness, and embarrassment.

Many critically examined their counselling practice of recommending specific amounts of exercise after having an embodied sense of the practical and emotional reality of living with obesity. Residents wrote about how they came to realize that their recommendations to patients might have been unrealistic and unhelpful. Most concluded that this experience allowed them to feel more empathetic to their patients.

Two residents felt disoriented as to the purpose of the empathy suit session and perceived it as ineffective and a waste of time.

### Reflexivity: examining assumption to improve practice

Learning about the complexity and chronicity of obesity encouraged residents to re-investigate their assumptions about the causes of obesity, management and counselling, and their professional identity with regards to supporting patients. For the majority, this reflection led to forming intentions to adopt more empathetic and comprehensive approaches to weight management.

The narratives illustrated a wide range of beliefs about and attitudes toward people with obesity that affect residents’ counselling practice. Some described their “personal frustration with being unable to help them manage their obesity” (participant 12). Others explained their difficulties accepting obesity as a disease (participant 22) and postulated that “in terms of science and numbers, it is possible for every single patient to lose weight” (participant 34). However, many described a shift in their knowledge and a re-thinking of their previously held assumptions resulting from the course. For example, residents described how lack of awareness of physiological and medical barriers to losing weight may have led to inappropriate weight loss expectations. Some reflected on the psychological impact that clinic environment or procedures, such as ill-fitting gowns or larger blood pressure cuffs, has on patients with obesity.

Furthemore, many explained how the course helped them recognize the important role they play in helping patients understand the complex factors contributing to weight, finding realistic strategies to improve health, and supporting them throughout their efforts. Others emphasized that they now recognized the importance of contextual factors of patients’ life history and circumstances. Many highlighted learning about prevention as a crucial part of their role as physicians.

Again, a small number of residents questioned the importance of the topic and were not open to reflect on their practice.

### The 5As and 5AsT tools: supported confidence

Most residents described the 5As of obesity management as a useful framework, and the 5AsT tools as helpful, to improve the quality of their practice and increase their confidence with weight counselling.

Almost all residents applied the 5As approach during their in-clinic practice. Many highlighted the importance of beginning the the conversation by asking the patient for permission to talk about weight. As a result, they felt they were able to create a respectful relationship with patients; and patients were more open to the discussion. Others emphasized the benefit of asking the patient about their story of weight gain for comprehensively assessing root causes. A number reflected on how the 5As approach requires practice, a long-term physician-patient relationship, and repeated follow-up encounters.

Many felt that using the 5As approach and tools in clinic allowed them to feel more comfortable with discussing weight and to experience more successful encounters. With these positive experiences, residents imagined themselves playing a positive role in supporting patients with obesity. Many expressed their intention to use the 5As for obesity management in their own practice, to adjust them to their patients’ needs, and refine their skill in using the approach.

### Complexity of obesity: challenges for practice

Narratives reflected how residents’ own experiences are enmeshed with societal values and beliefs about obesity, which can pose challenges in their encounters with patients.

Some described discomfort with the subject and fear of offending patients. Others wrote about how they perceived patients to “fail” with weight management and, as a result, feel frustrated with being unable to help. Residents described how they noticed themselves judging patients’ motivation or intelligence, feeling challenged by patients’ questions, frustrated, and questioning the utility of weight counselling all together. Time limitations were mentioned as another challenge. A small number explained the difficulty of letting go of expectations of weight loss for both patients and for themselves as physicians.

Many of these reflections on challenges demonstrate that obesity is often perceived as a product of the patients’ lifestyle and personal qualities.

## Discussion

The 5AsT-MD course proved a promising approach to better prepare residents for obesity counselling. The course improved residents’ knowledge, confidence, and attitudes to engage in patient-centred conversations about obesity. The study findings demonstrate the overall success of the course, yet they also reveal the unique challenges of teaching obesity management in residency programs. These challenges highlight precisely why there is a need for obesity prevention and management training in family medicine. Due to insufficient training in medical school and lack of understanding of the disease, an over-simplified approach may perpetuate unsupportive blame and shame. While most residents considered obesity prevention and management an important aspect of their work, less than a third felt they had received sufficient training. After the course about half felt better prepared and a large majority felt inspired to learn more about the subject in order to improve their practice.

Using a multi-methods approach provided a richer understanding of how the content and pedagogical approach of the 5AsT-MD course impacted learners. Findings elucidate that understanding the complexity and chronicity of obesity is important for developing empathy. Experiential learning components of the course were particularly impactful and facilitated critical reflection that served to enhance professional identity development around non-judgemental and compassionate care of patients living with obesity.

Medical education has not responded adequately to the complexity of obesity and the need for tailored coordinated primary care. As a result, individual physicians’ approach to obesity largely focuses on treating comorbidities and counselling lifestyle changes such as diet and exercise. As our findings confirm, this approach is often ineffective and leads both the patient and the physician to have unrealistic expectations, feel frustration, and contribute to feelings of shame and blame [14]. Avoiding stigmatization in health care, as well as addressing the psychological aspects of living with obesity, have been emphasized as one of the most important tasks for primary care obesity management in the recently developed guidelines for patient-centred obesity management in Europe [45].

Because of the entanglement of obesity in societal attitudes, merely increasing didactic teaching of obesity content is not sufficient. Our results demonstrate that residents need support to examine the assumptions that underlie their practice and transform their approach to be more evidence-based and patient-centred. There are calls for educational approaches that will help address the growing inequalities within health care systems worldwide. Just as the Flexner report changed the landscape of medical education in North America in the twentieth century [46], the Lancet Commission Report is raising the need for transformative learning as an approach that will support global complexities while responding to local contextualities [42]. Transformative pedagogy is about inviting “deep structural shifts” in thinking [47]. by providing learners with the the opportunities to reframe their thinking and meaning structures in order to alter their behaviours, attitudes, and beliefs [48, 49]. In a recent scoping review [50]. transformative learning in health professional education was found to occur when: learners critically engaged with their beliefs, biases, and habits of mind; learners were encouraged to become independent, reflective, and critical thinkers; learners were immersed in different contexts, specifically outside of the classroom; learners were exposed to interactive and experiential learning; and, learners practiced competencies that supportted new ways of thinking (i.e., empathetic listening). Importantly, this review outlined that transformative learning has been shown to influence professional identity development by heightening the awareness of others and enhancing humanistic values, key components of delivering quality and safe care to patients and families. The empathy suit experience was designed for this purpose and allowed residents to confront their assumptions about persons living with obesity and expose the difficulties involved with routine daily activities. Our findings suggest such an approach can help foster critical consciousness [51] —a reorientation of perspective towards social justice—that can “rehumanize” relationships and improve patient care.

While the 5AsT-MD course resulted in a shift towards a more empathic, engaged, and comprehensive approach, the outcomes varied depending on beliefs and attitudes of individual residents prior to the course. It is important to “scaffold” the information when designing obesity training and build from where the learner audience is at [52]. The narratives reflected how emotionally laden the topic is for many. As a result, there is a danger to create a level of discomfort and disorientation that hinders reflection and learning. On the other hand, discomfort and embodied experiences can facilitate learning about how to improve practice [53]. Findings underscore the need for further research on effective pedagogical strategies for training courses focused on improving the confidence, knowledge, and attitudes of family medicine residents working with people with obesity. Disseminating and adapting the course to various different educational contexts requires drawing on research on different modes of delivery including, online resources, that allow for experiential and transformative learning [54].

Overall, residents greatly appreciated the 5As of obesity management as a framework and the 5AsT tools to guide them through their conversation with the patient.

### Limitations

While the sample size is small comparatively, the course took place in a large residency program in Canada and, therefore, we feel reflects a valid sample with findings that will be of interest and practicality for medical educators. Mean scores for both the BAOP and ATOP were higher than means found in other studies [43, 48, 49, 51]. This may suggest that residents may not have felt comfortable expressing negative views for fear that they would be evaluated poorly. However, narrative reflections contained a number of negative responses suggesting that residents felt free to express their opinion.

## Conclusions

Tailored and co-ordinated primary care is crucial to reducing obesity and improving health. 5AsT-MD has the potential as a course for increasing residents’ knowledge of obesity and its complexity, as well as their competency and confidence in engaging patients in effective obesity management. The course’ pedagogical orientation and experiential components offer a novel approach to obesity management training that stretches beyond the biomedical realm and introduces the human complexity and contextuality of living with obesity. This study illustrates how this course fostered transformative learning through engaging learners in experiences offered spaces to reflect and think about what it is like to live with obesity. Our results inform an ongoing process of further refining and disseminating the course to other institutions.

## Supplementary information

**Additional file 1.** Pre/post Workshop Assessment.

**Additional file 2.** Narrative Reflection Instructions.

## Data Availability

The datasets used and/or analysed during the current study are available from the corresponding author on reasonable request.

## Abbreviations

5As: 5As of Obesity Management™: ASK, ASSESS, ADVISE, AGREE, ASSIST
5AsT: 5As Team research program
5AsT-MD: 5AsT for Doctors of Medicine
ATOP: Attitudes Towards Obese Persons Scale
BAOP: Beliefs About Obese Persons Scale
DPR: Doctor-Patient Relationship course
