# Supplementary material for: Improving obesity management training in family medicine: multi-methods evaluation of the 5AsT-MD pilot course
Source: BMC Med Educ. 2020 Jan 7;20:5. doi: 10.1186/s12909-019-1908-0 (PMC6947955; doi:10.1186/s12909-019-1908-0)
Supplement: Supplementary file 1 — Additional file 1. Pre/post Workshop Assessment. [file 12909_2019_1908_MOESM1_ESM.docx]

Additional file 1: Pre/Post Workshop Questionnaire


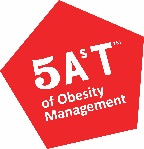


For Office Use Only

Participant #: _______

**Obesity Management – Pre/Post Workshop Assessment**

Completion of this questionnaire is mandatory for the Doctor-Patient Relationship course. If you consented to participate in the 5AsT study, your responses will be completely de-identified prior to being given to the research team.

**Date (mm/dd/year):** **______________________________________**

**How old are you?**

- 20-25 years old
- 26-30 years old
- 31-35 years old
- 36-40 years old
- 40+ years old

**What gender do you identify as?**

- Female
- Male
- Other

**How many years of medical training have you completed (including medical school, residency, and fellowships)?**

- 3 years
- 4 years
- 5 years
- 6+ years

**Please indicate your agreement with the following statements by placing an “X” in the appropriate box.**

|  | Strongly Agree | Agree | Neutral | Disagree | Strongly Disagree |
| --- | --- | --- | --- | --- | --- |
| I believe that obesity management is an important part of my job as a family physician. | ☐ | ☐ | ☐ | ☐ | ☐ |
| My medical training before this session has adequately prepared me to understand and manage obesity with patients. | ☐ | ☐ | ☐ | ☐ | ☐ |
| I am motivated to learn more about the effective prevention and management of obesity. | ☐ | ☐ | ☐ | ☐ | ☐ |

**Please rate how comfortable you are doing the following. Please check the one that most applies.**

|  | Very Comfortable | Somewhat Comfortable | Neither Comfortable nor Uncomfortable | Somewhat Uncomfortable | Very Uncomfortable |
| --- | --- | --- | --- | --- | --- |
| Asking for a patient’s permission to talk about his/her weight. | ☐ | ☐ | ☐ | ☐ | ☐ |
| Assessing a patient’s obesity-related risks and complications. | ☐ | ☐ | ☐ | ☐ | ☐ |
| Assessing a patient’s potential root causes of weight gain. | ☐ | ☐ | ☐ | ☐ | ☐ |
| Advising patients on obesity-related risks and complications. | ☐ | ☐ | ☐ | ☐ | ☐ |
| Advising patients on available treatment options for obesity. | ☐ | ☐ | ☐ | ☐ | ☐ |
| Advising patients on long-term strategies to manage weight. | ☐ | ☐ | ☐ | ☐ | ☐ |
|  | Very Comfortable | Somewhat Comfortable | Neither Comfortable nor Uncomfortable | Somewhat Uncomfortable | Very Uncomfortable |
| Agreeing with patients on realistic weight-loss expectations. | ☐ | ☐ | ☐ | ☐ | ☐ |
| Agreeing with patients on sustainable behavioural/lifestyle goals. | ☐ | ☐ | ☐ | ☐ | ☐ |
| Agreeing with patients on goals for health outcomes. | ☐ | ☐ | ☐ | ☐ | ☐ |
| Assisting patients in addressing their barriers to proper weight management. | ☐ | ☐ | ☐ | ☐ | ☐ |
| Providing education and resources to encourage patients’ self-management. | ☐ | ☐ | ☐ | ☐ | ☐ |
| Counseling patients on physical activity and weight control. | ☐ | ☐ | ☐ | ☐ | ☐ |
| Counseling patients on appropriate weight gain during pregnancy. | ☐ | ☐ | ☐ | ☐ | ☐ |
| Counseling patients on emotional eating. | ☐ | ☐ | ☐ | ☐ | ☐ |
| Counseling patients on weight-related depression and anxiety. | ☐ | ☐ | ☐ | ☐ | ☐ |
| Counseling patients on iatrogenic causes of weight gain. | ☐ | ☐ | ☐ | ☐ | ☐ |
| Counseling patients who have children with obesity. | ☐ | ☐ | ☐ | ☐ | ☐ |
| Addressing differences that may come up in your consultation due to culture or beliefs. | ☐ | ☐ | ☐ | ☐ | ☐ |
| Addressing weight gain with patients who have multiple co-morbidities. | ☐ | ☐ | ☐ | ☐ | ☐ |
| Discussing weight with patients who have a family history of obesity. | ☐ | ☐ | ☐ | ☐ | ☐ |
| Discussing weight and lifestyle management with patients who are at risk of obesity. | ☐ | ☐ | ☐ | ☐ | ☐ |
| Referring patients with obesity to the appropriate healthcare provider for care (i.e. mental health worker, dietician, exercise specialist, bariatric specialist). | ☐ | ☐ | ☐ | ☐ | ☐ |

**Are there any other aspects of obesity management that you are uncomfortable or feel unconfident doing in practice?**

**At this point in your training, would you be confident enough to incorporate obesity management into your medical practice?**

**Do you have any additional comments?**
