# Supplementary material for: Improving obesity management training in family medicine: multi-methods evaluation of the 5AsT-MD pilot course
Source: BMC Med Educ. 2020 Jan 7;20:5. doi: 10.1186/s12909-019-1908-0 (PMC6947955; doi:10.1186/s12909-019-1908-0)
Supplement: Supplementary file 2 — Additional file 2. Narrative Reflection Instructions. [file 12909_2019_1908_MOESM2_ESM.docx]

Additional file 2: Narrative Reflection Instructions

| 1. **Post Bariatric Suit Experience** | Please reflect on your experience of wearing the bariatric suit. |
| --- | --- |
|  | Include any thoughts, feelings, bodily sensations triggered, and insights gained, by wearing the suit, performing activities, or interacting with others in the smart condo. |
| 1. **Post Patient Visit** | Please reflect on your experience of a weight-management conversation using the 5As framework with one of your patients. |
|  | Include any thoughts, feelings, challenges, successes, insights, or questions you may have experienced during or after the conversation with regards to yourself as a family physician, your approach to weight management, to your patient, or to the utility of the 5As. |
